# Supplementary material for: A Humanized Yeast Phenomic Model of Deoxycytidine Kinase to Predict Genetic Buffering of Nucleoside Analog Cytotoxicity
Source: Genes (Basel). 2019 Sep 30;10(10):770. doi: 10.3390/genes10100770 (PMC6826991; doi:10.3390/genes10100770)
Supplement: Supplementary file 1 [file genes-10-00770-s001.zip › Additional_Files/Additional_File7_GOTermHeatmaps.zip!/Additional_File7_GOTermHeatmaps/small_nuclear_ribonucleoprotein_complex.pdf]

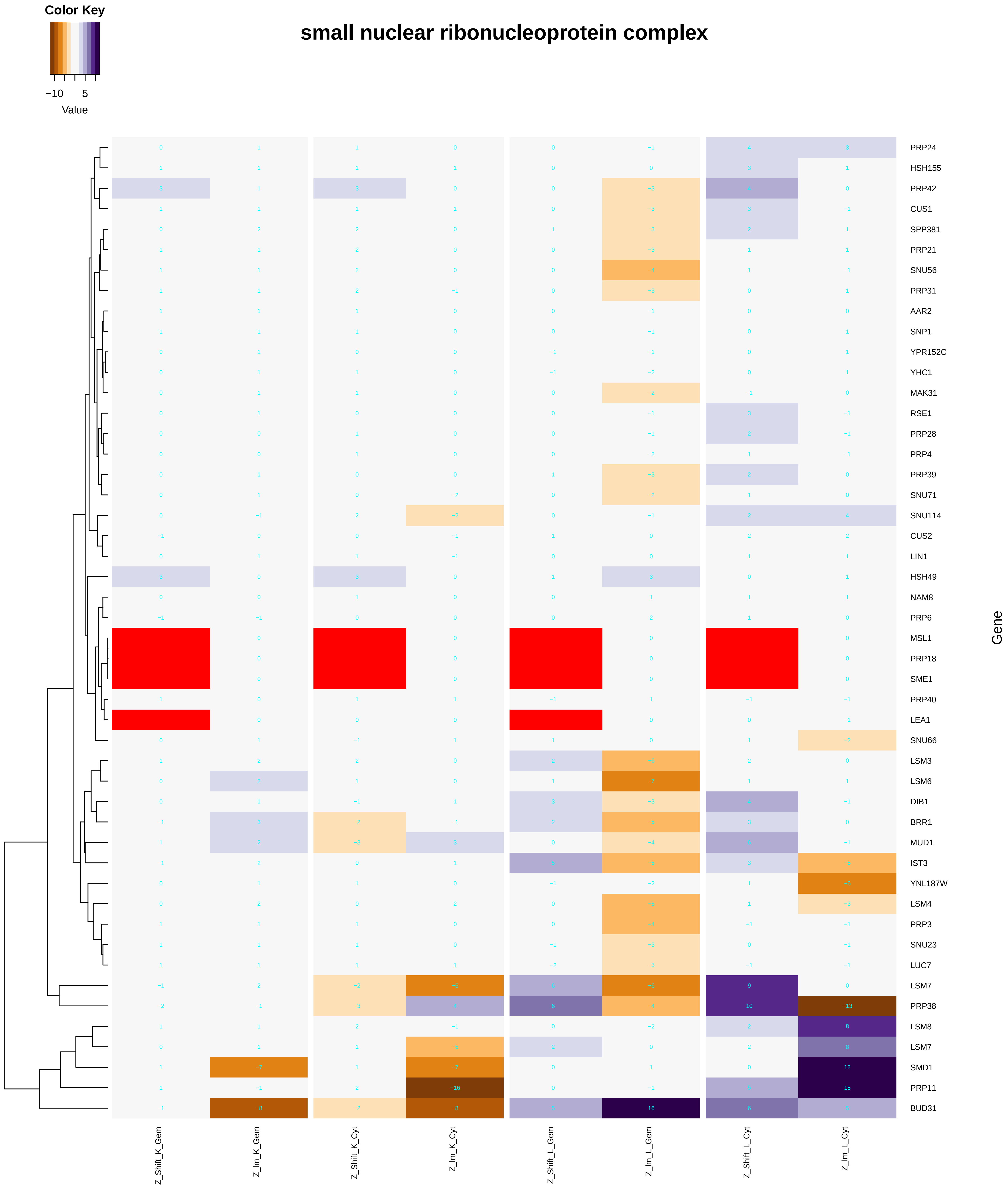
small nuclear ribonucleoprotein complex
Color Key
−10
5
Value
PRP24
HSH155
PRP42
CUS1
SPP381
PRP21
SNU56
PRP31
AAR2
SNP1
YPR152C
YHC1
MAK31
RSE1
PRP28
PRP4
PRP39
SNU71
SNU114
CUS2
LIN1
HSH49
NAM8
PRP6
MSL1
PRP18
SME1
PRP40
LEA1
SNU66
LSM3
LSM6
DIB1
BRR1
MUD1
IST3
YNL187W
LSM4
PRP3
SNU23
LUC7
LSM7
PRP38
LSM8
LSM7
SMD1
PRP11
BUD31
Gene
Z_Shift_K_Gem
Z_Im_K_Gem
Z_Shift_K_Cyt
Z_Im_K_Cyt
Z_Shift_L_Gem
Z_Im_L_Gem
Z_Shift_L_Cyt
Z_Im_L_Cyt

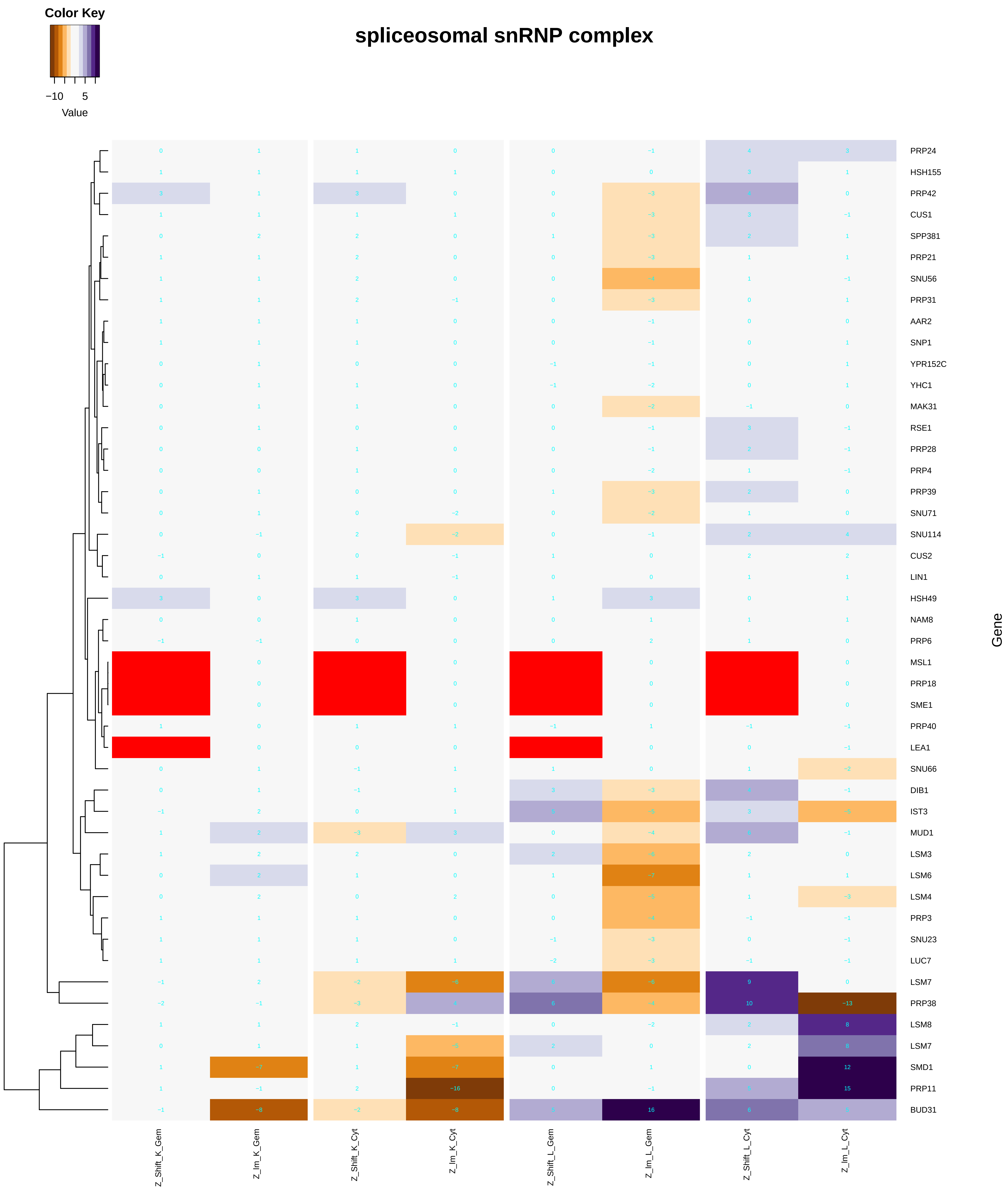

spliceosomal snRNP complex
Color Key
−10 5
Value
PRP24
HSH155
PRP42
CUS1
SPP381
PRP21
SNU56
PRP31
AAR2
SNP1
YPR152C
YHC1
MAK31
RSE1
PRP28
PRP4
PRP39
SNU71
SNU114
CUS2
LIN1
HSH49
NAM8
PRP6
MSL1
PRP18
SME1
PRP40
LEA1
SNU66
DIB1
IST3
MUD1
LSM3
LSM6
LSM4
PRP3
SNU23
LUC7
LSM7
PRP38
LSM8
LSM7
SMD1
PRP11
BUD31
Gene
Z_Shift_K_Gem
Z_Im_K_Gem
Z_Shift_K_Cyt
Z_Im_K_Cyt
Z_Shift_L_Gem
Z_Im_L_Gem
Z_Shift_L_Cyt
Z_Im_L_Cyt

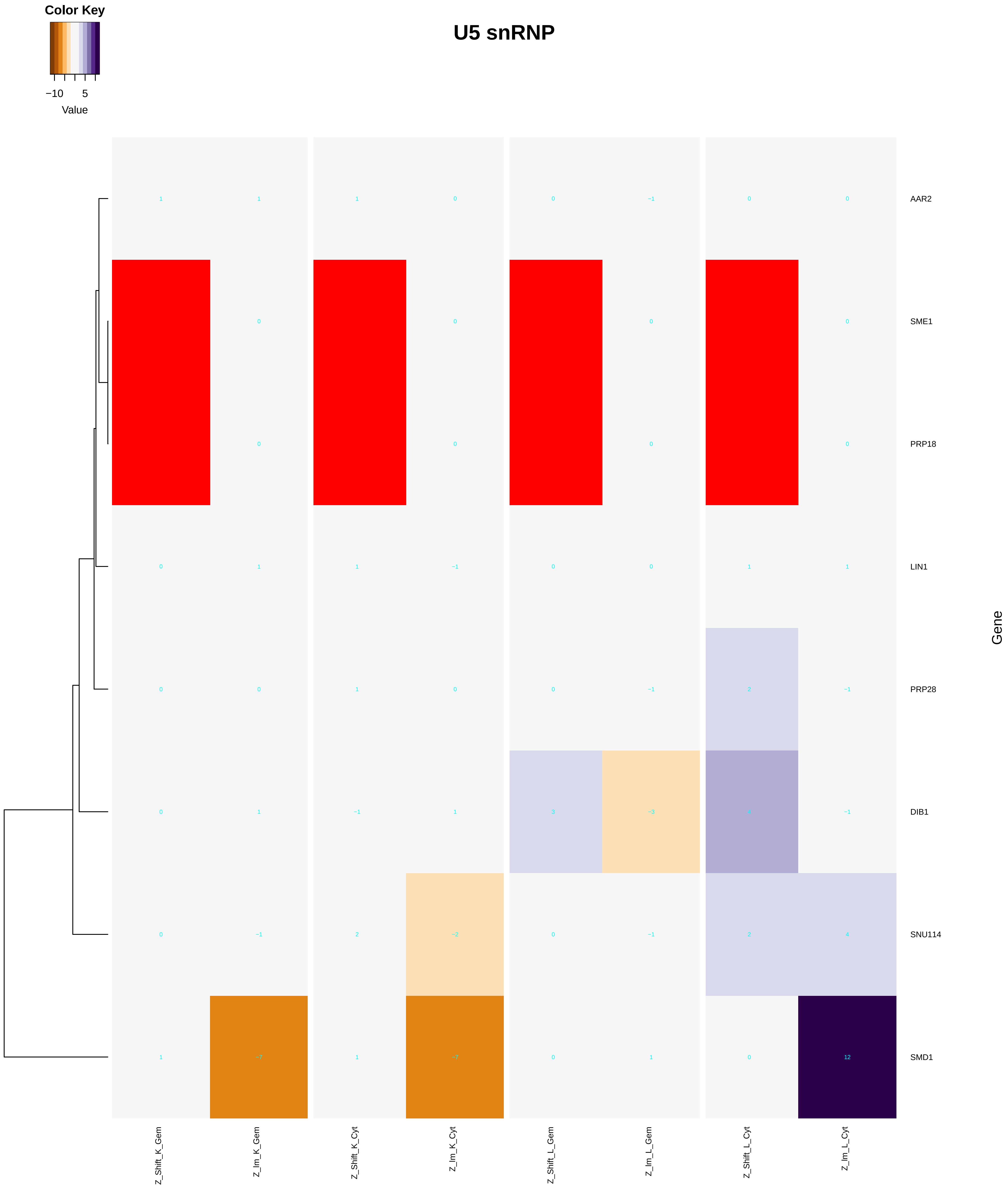
Color Key
−10
5
Value
U5 snRNP
AAR2
SME1
PRP18
LIN1
PRP28
DIB1
SNU114
SMD1
Gene
Z_Shift_K_Gem
Z_Im_K_Gem
Z_Shift_K_Cyt
Z_Im_K_Cyt
Z_Shift_L_Gem
Z_Im_L_Gem
Z_Shift_L_Cyt
Z_Im_L_Cyt

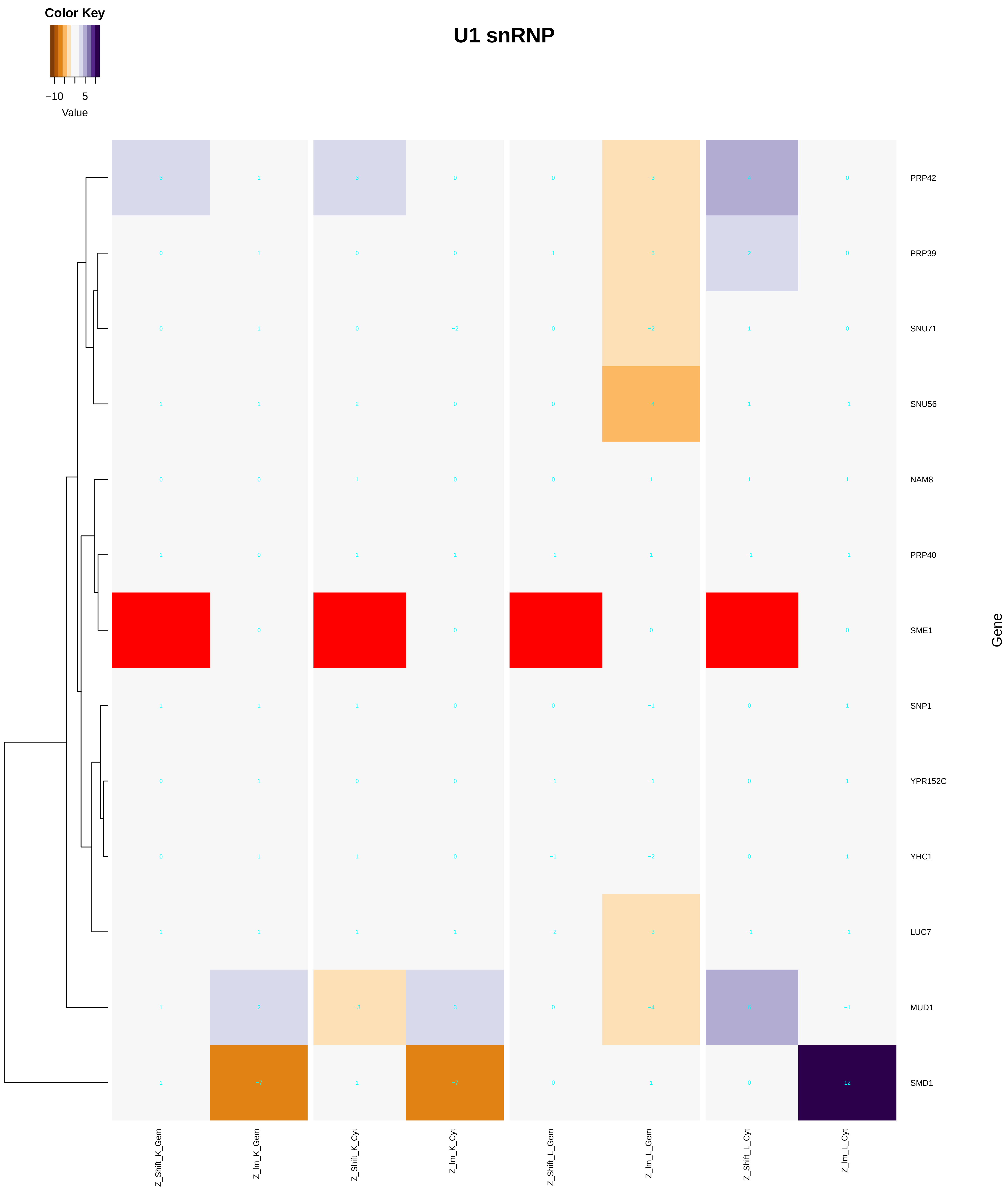

U1 snRNP
Color Key
−10
5
Value
PRP42
PRP39
SNU71
SNU56
NAM8
PRP40
SME1
SNP1
YPR152C
YHC1
LUC7
MUD1
SMD1
Gene
Z_Shift_K_Gem
Z_Im_K_Gem
Z_Shift_K_Cyt
Z_Im_K_Cyt
Z_Shift_L_Gem
Z_Im_L_Gem
Z_Shift_L_Cyt
Z_Im_L_Cyt

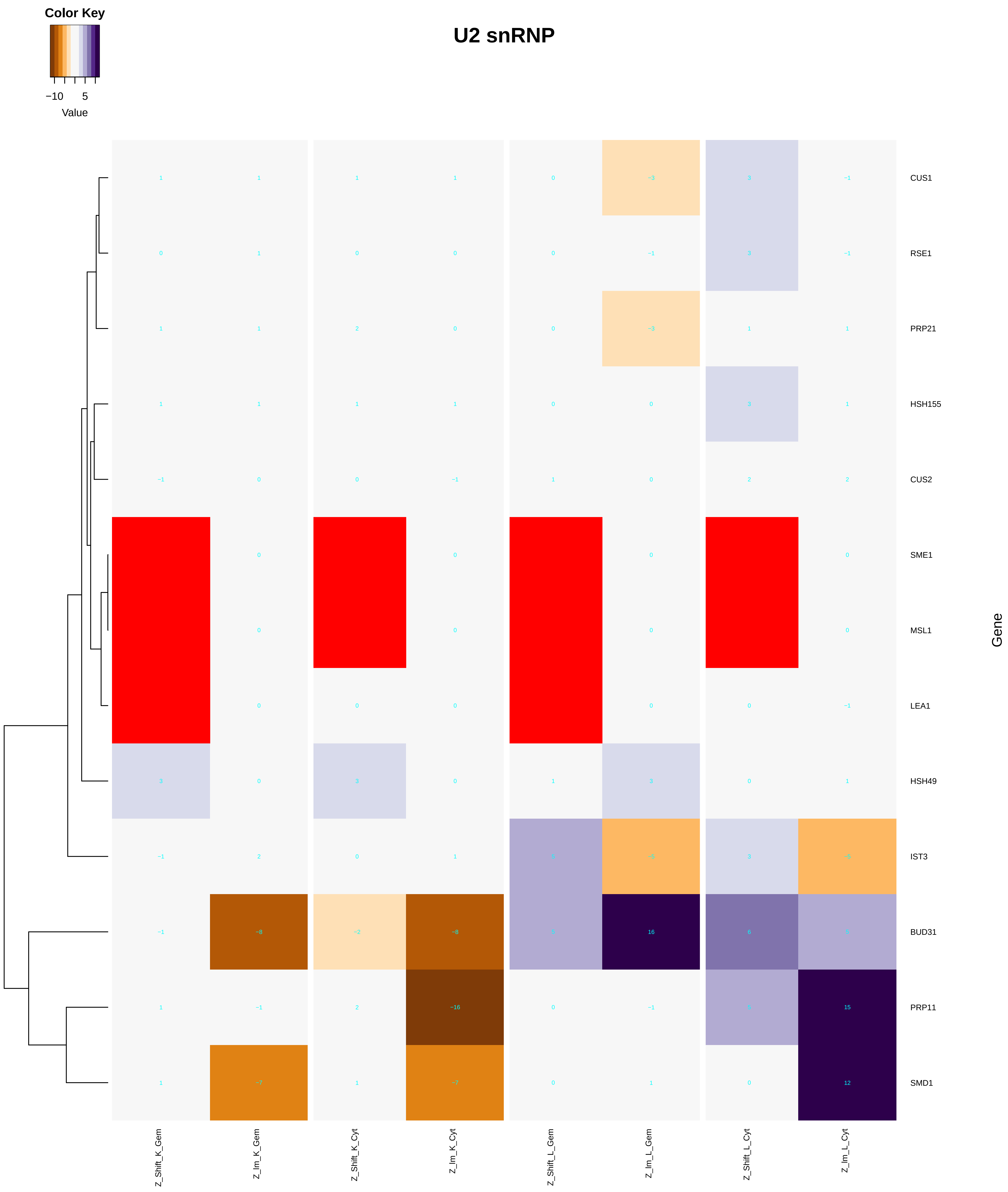

Color Key
−10
5
Value
U2 snRNP
CUS1
RSE1
PRP21
HSH155
CUS2
SME1
MSL1
LEA1
HSH49
IST3
BUD31
PRP11
SMD1
Gene
Z_Shift_K_Gem
Z_Im_K_Gem
Z_Shift_K_Cyt
Z_Im_K_Cyt
Z_Shift_L_Gem
Z_Im_L_Gem
Z_Shift_L_Cyt
Z_Im_L_Cyt

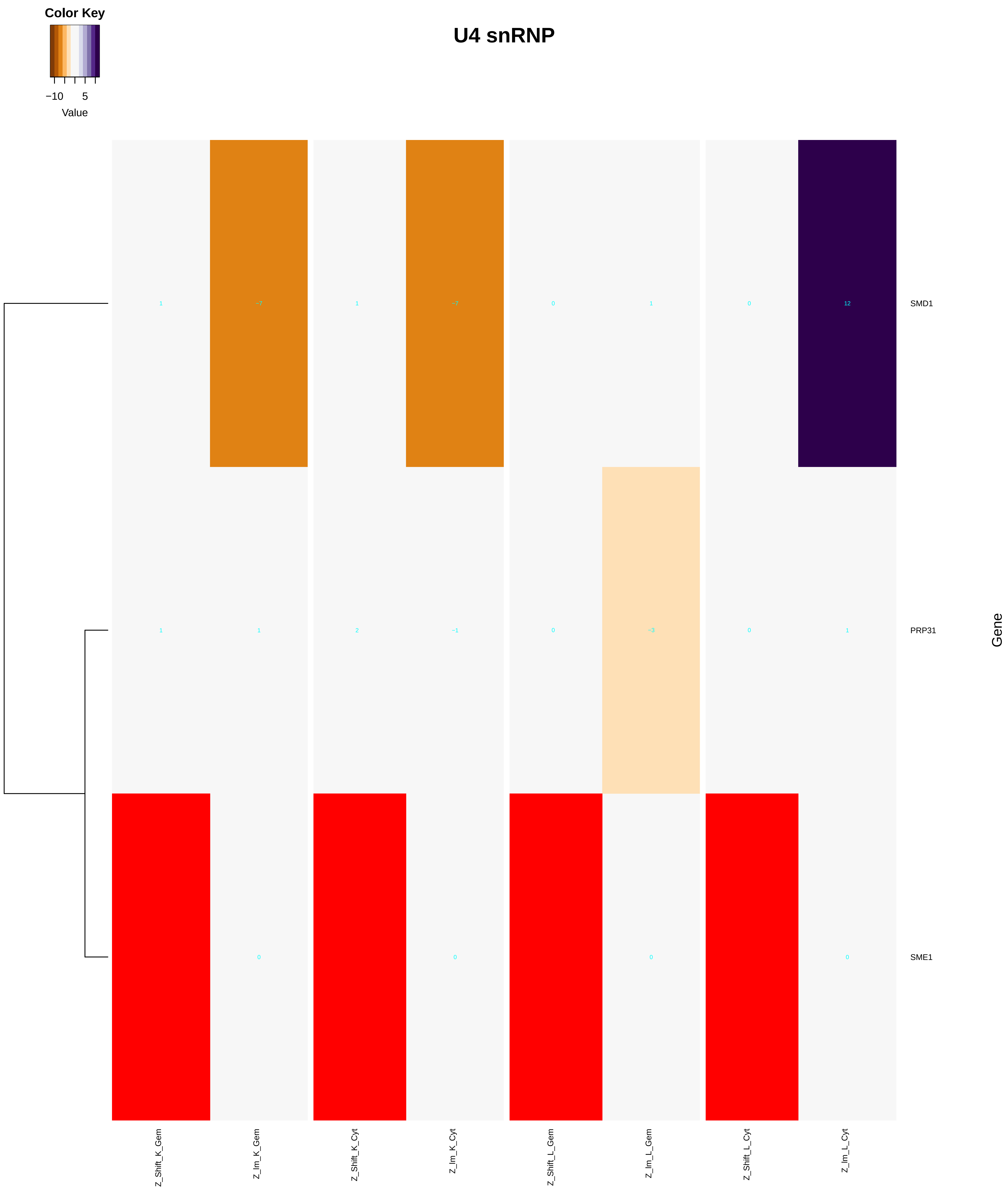
Color Key
−10
5
Value
U4 snRNP
1
−7
1
−7
0
1
0
12
SMD1
1
1
2
−1
0
−3
0
1
PRP31
0
0
0
0
SME1
Gene
Z_Shift_K_Gem
Z_Im_K_Gem
Z_Shift_K_Cyt
Z_Im_K_Cyt
Z_Shift_L_Gem
Z_Im_L_Gem
Z_Shift_L_Cyt
Z_Im_L_Cyt

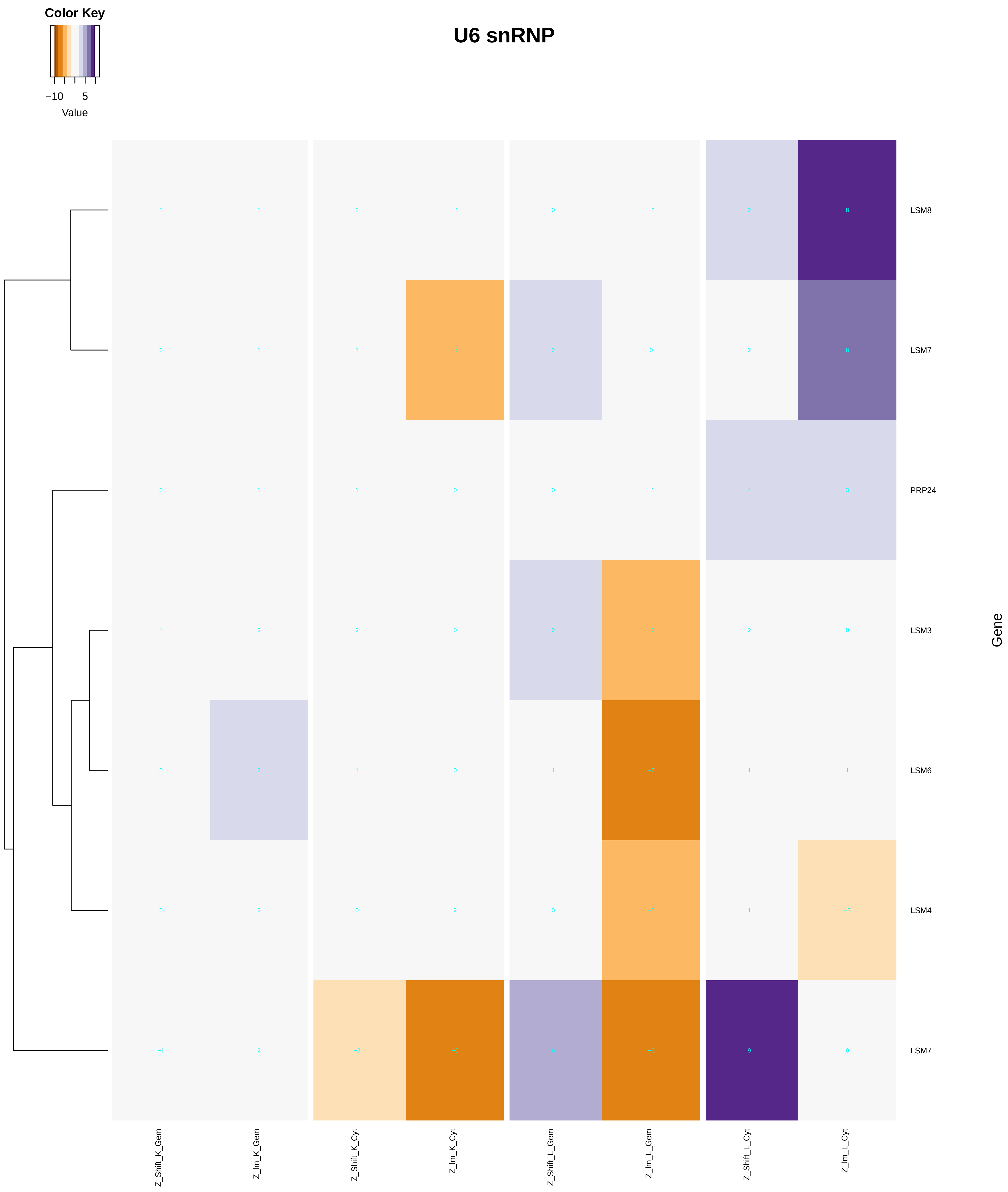
U6 snRNP
Color Key
−10
5
Value
LSM8
LSM7
PRP24
LSM3
LSM6
LSM4
LSM7
Gene
Z_Shift_K_Gem
Z_Im_K_Gem
Z_Shift_K_Cyt
Z_Im_K_Cyt
Z_Shift_L_Gem
Z_Im_L_Gem
Z_Shift_L_Cyt
Z_Im_L_Cyt

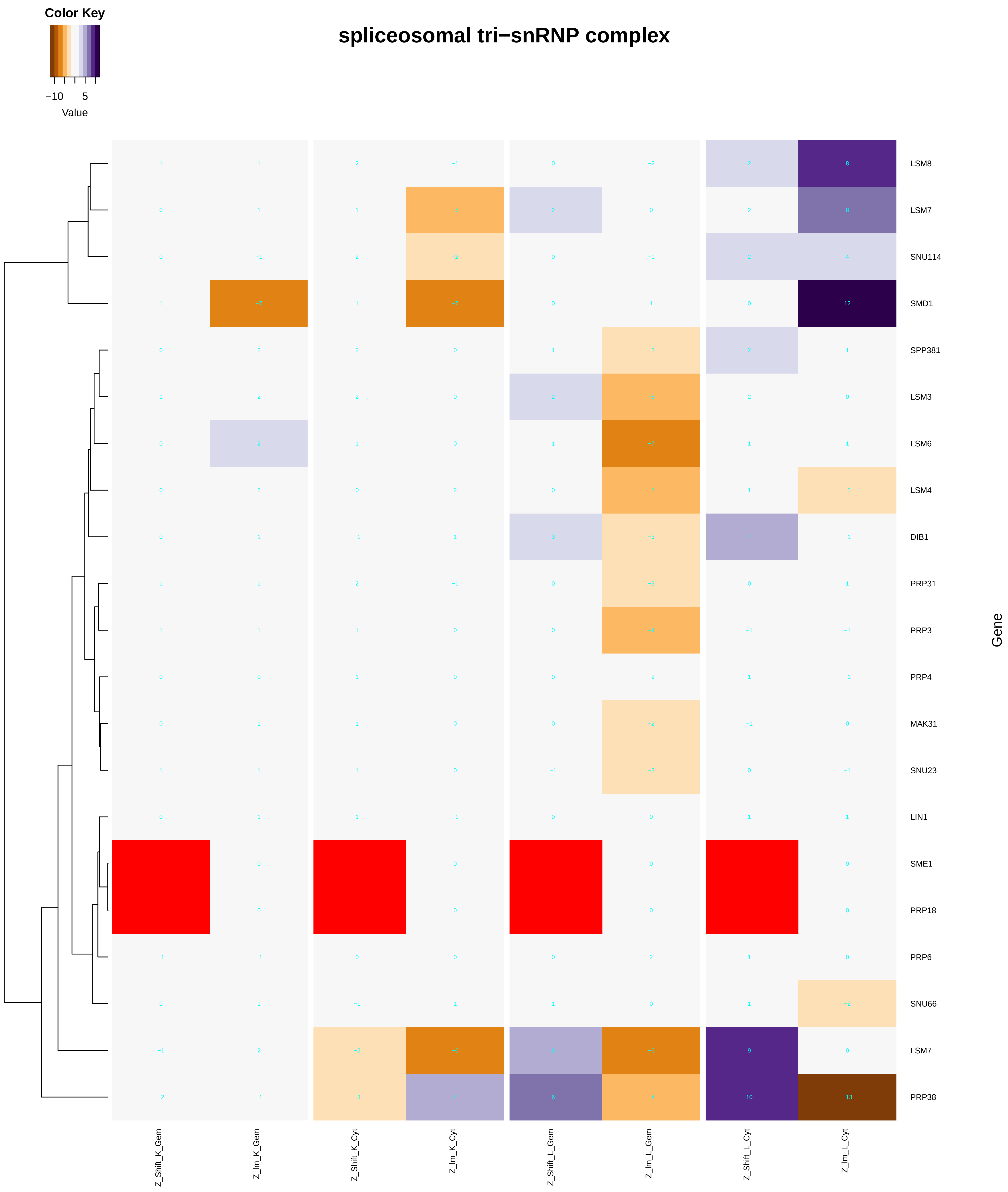
Color Key
−10
5
Value
spliceosomal tri−snRNP complex
LSM8
LSM7
SNU114
SMD1
SPP381
LSM3
LSM6
LSM4
DIB1
PRP31
PRP3
PRP4
MAK31
SNU23
LIN1
SME1
PRP18
PRP6
SNU66
LSM7
PRP38
Gene
Z_Shift_K_Gem
Z_Im_K_Gem
Z_Shift_K_Cyt
Z_Im_K_Cyt
Z_Shift_L_Gem
Z_Im_L_Gem
Z_Shift_L_Cyt
Z_Im_L_Cyt

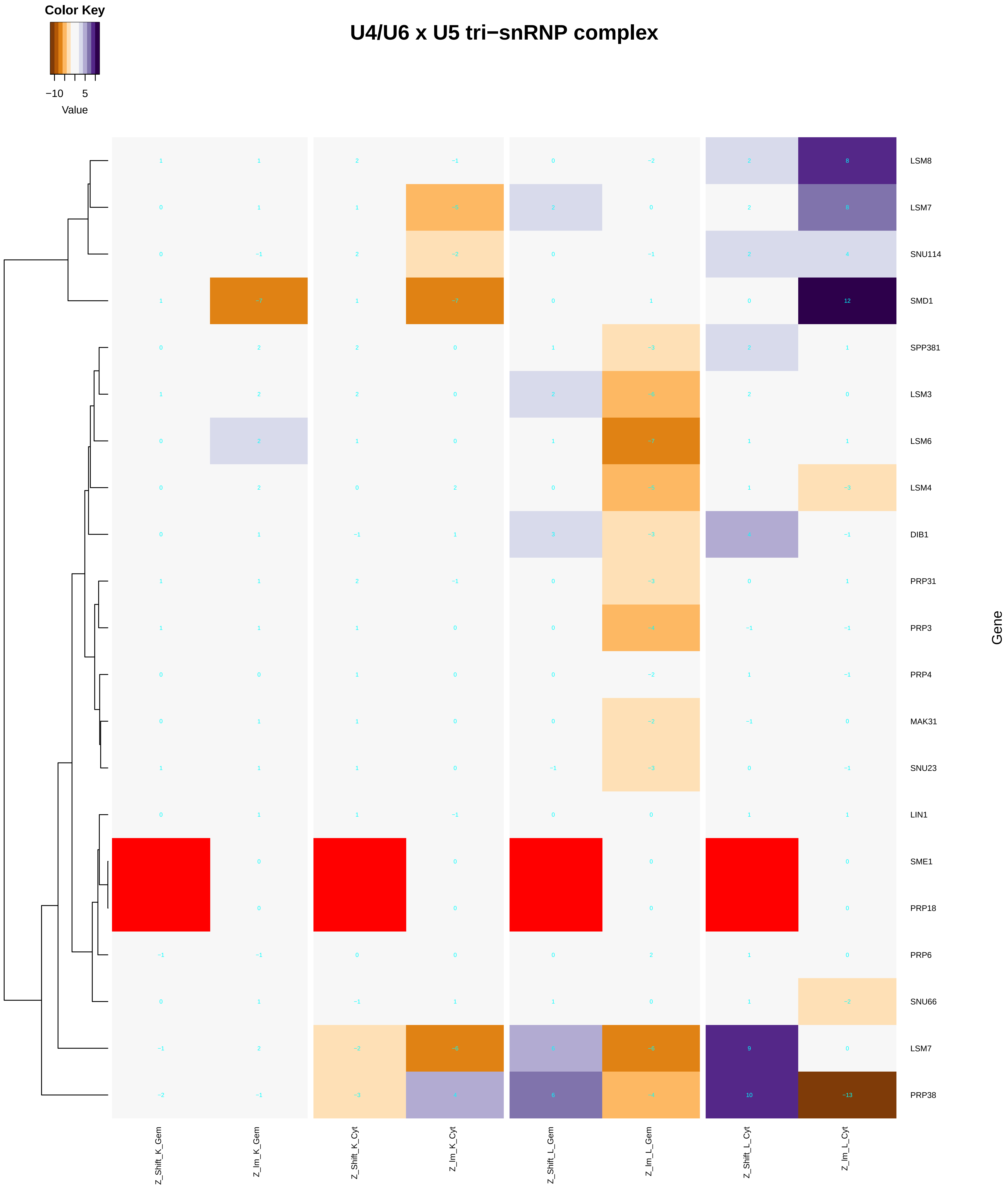

Color Key
−10
5
Value
U4/U6 x U5 tri−snRNP complex
LSM8
LSM7
SNU114
SMD1
SPP381
LSM3
LSM6
LSM4
DIB1
PRP31
PRP3
PRP4
MAK31
SNU23
LIN1
SME1
PRP18
PRP6
SNU66
LSM7
PRP38
Gene
Z_Shift_K_Gem
Z_Im_K_Gem
Z_Shift_K_Cyt
Z_Im_K_Cyt
Z_Shift_L_Gem
Z_Im_L_Gem
Z_Shift_L_Cyt
Z_Im_L_Cyt
